# Understanding the impact of dementia on spousal relationships: A qualitative study with female spousal carers of people living with dementia

**DOI:** 10.1177/14713012241286559

**Published:** 2024-09-27

**Authors:** Elien Van Hout, Milena Contreras, Eneida Mioshi, Naoko Kishita

**Affiliations:** School of Health Sciences, 6106 University of East Anglia, UK; School of Philosophy, Psychology and Language Sciences, 120061 University of Edinburgh, UK; School of Health Sciences, 6106 University of East Anglia, UK

**Keywords:** Alzheimer’s disease, marital relationship, interaction, intimacy, mental health

## Abstract

**Background:** Dementia does not merely affect individuals, the carer and the person living with dementia, but also has a profound impact on their spousal relationship. As such, this study aimed to gain a deeper understanding of how dementia affects spousal relationships with a focus on interpersonal (i.e. relationship adjustment, communication engagement and emotional connection between two individuals) and intrapersonal (i.e. loss of self within the context of relationships) dynamics using a qualitative approach. The study also explored how carers adapt to such relationship challenges in the context of dementia care.

**Methods:** A phenomenological approach was used to capture the subjective experiences of female spousal carers, who regularly support their partner living with dementia. A total of nine semi-structured interviews were conducted.

**Results:** *Relationship adjustment* theme highlighted how learning to acknowledge role shifts from a spouse to a carer is critical for carers to manage relationship difficulties. *Emotional connection* theme demonstrated the importance of reminiscing about the shared history between dyads to cope with feelings of loss of affective intimacy. *Communication engagement* theme revealed carers’ need to learn a new way of communicating due to the decrease in meaningful communication and two-way interaction. *Sense of self* theme highlighted the importance of self-compassion to overcome feelings of self-loss and isolation.

**Conclusion:** Findings suggest that improving the relationship between female spousal carers and their partner living with dementia may require targeted interventions addressing different factors. Such interventions can include a couple’s life story approach to enable couples to reminisce about their shared experiences, interactive communication training to enhance meaningful engagements, and a psychological approach such as compassion-focused therapy to overcome emotional challenges and facilitate self-compassion.

## Introduction

In the UK, approximately 920,000 people are living with dementia and this prevalence rate is expected to rise even further in the future (Alzheimer’s Society, 2019). By 2050, it is expected that more than 131 million people will have dementia worldwide (Xiong et al., 2020). Most people with dementia in the UK live at home and 80% of these people are supported by unpaid family carers, with the majority being older women (Alzheimer’s Society, 2019).

Given the complex situation of caregiving, often both negative and positive feelings emerge when carers are asked about their experiences. Positive aspects of experiences include an increase in carers’ self-esteem, a stronger bond with the care recipient, and an increase in meaning in life (Frias et al., 2020). However, being a carer for a person living with dementia is a complex situation which may also negatively impact the carer’s mental, physical, and social functioning (Stall et al., 2019).

According to Kitwood (1997), dementia does not merely affect individuals but rather has a profound impact on relationships. The act of caregiving occurs within the context of a relationship, and this relationship plays a crucial role in both, the formation and continuation of caregiving (McGovern, 2011). For example, a qualitative study exploring how motivation and relationship dynamics influence carers’ subjective experience of dementia caregiving, found that carers’ capacity to find meaning in their role and relationship with the care recipient appears to affect the motivation to continue providing good care (Quinn et al., 2015).

One concept extensively examined and evaluated in recent literature is couplehood, defined as the dynamic partnership between spouses in the face of dementia (Weicht & Tolhurst, 2023). Couplehood illuminates the significance of an unified approach to caregiving and maintaining involvement of both partners (Stedje et al., 2023; Weicht & Tolhurst, 2023). In addition, many theories within the dementia caregiving context, such as the grief-stress model of caregiving (Noyes et al., 2010), highlight the importance of considering other interpersonal (i.e. relationship adjustment, communication engagement, and emotional connection between two individuals) and intrapersonal dynamics (i.e. loss of self within the context of relationship) when exploring the healthy relationship (Knobloch et al., 2019; Lyons & Lee, 2018; Rippon et al., 2020; Stedje et al., 2023). Nevertheless, these dynamics are still somewhat underexplored empirically in the current dementia literature compared to other factors that can affect carer experiences (e.g. coping skills) since such dynamics are often challenging to capture quantitatively (Knobloch et al., 2019). Therefore, this study aims to gain a deeper understanding of these interpersonal and intrapersonal dynamics using a qualitative approach and explore these different dynamics in terms of meaning given to them by family carers of people living with dementia. A meta-analysis of studies comparing carer spouses, adult children, and children-in-law, concluded that spousal carers report greater burden, lower levels of psychological wellbeing, and greater strain on their relationship (Pinquart & Sörensen, 2011). Given that the majority of carers are women and spousal carers are more likely to be affected by the relationship strain, this study will focus on gaining insight into the meaning given to relationship dynamics among female spousal dementia carers.

### Interpersonal dynamics in spousal caregiving

The presence of successful relationship adjustment is considered to be one of the critical components of the healthy interpersonal dynamics between the carer and the care-recipient and positively associated with the wellbeing of carers (Hawken et al., 2018). In general, relationship adjustment is described as a combination of a good balance within their relationship experience and positive evaluations or attitudes toward this relationship and the partner (Fincham & Rogge, 2010). Equity theory suggests that couples generally strive to maintain a good balance of give and take in carer-partner relationships (Hatfield & Rapson, 2012). However, when a couple is faced with a serious illness, imbalance (inequity) can occur leading to negative attitudes and increased distress among both carer and the care recipient (Rippon et al., 2020; Shim et al., 2012).

Communication engagement also plays an important role in maintaining the heathy interpersonal dynamics. Communication engagement refers to how actively and explicitly people communicate with a partner (Knobloch et al., 2019). It is not simply about reaching an agreement but the presence of joint collaborative effort. This effort made by both carer and care recipient to engage and empathise, even when content is not understood, is critical for positive experiences of communication (Alsawy et al., 2020). Communication engagement is essential for sustaining valued relationships and the absence of engagement can lead to social isolation, loneliness, depression and anxiety (Wawrziczny et al., 2016).

A third critical component of the healthy interpersonal dynamics is the emotional connection carers and care recipients experience. Emotional connection refers to the feelings of bonding and being valued and understood by each other, which often involves the process of sharing various subjective feelings (Baumeister & Vohs, 2007). The presence of emotional connection or perceived closeness between two people is an important component of relationship quality or satisfaction (Enright et al., 2020). This connection tends to decrease in the carer-care recipient relationship after a dementia diagnosis, leaving the carer feeling isolated and potentially resulting in increased mental health difficulties (Alsawy et al., 2020; Enright et al., 2020).

### Intrapersonal dynamics in spousal caregiving

Carers often experience a role transition from partner/spouse to carer (Lee et al., 2019). Such transformed relationship is known to contribute to a loss of couple identity (Hernandez et al., 2017), potentially leading to a loss of identity in the carer themselves (Tuomola et al., 2016). Loss of self refers to perceived changes in “the individual’s characteristic ideas about who they are and what they are like” (Baumeister & Vohs, 2007). The loss of self is related to negative outcomes in carers of people living with dementia, including poorer wellbeing and mental health (Enright et al., 2020).

Investigating the subjective experiences of female spousal carers of people living with dementia with regard to these interpersonal and intrapersonal dynamics will allow for a deeper understanding of the meaning derived from these dynamics and how carers adapt to changes in such dynamics. The findings of this study will inform which inter- and intrapersonal dynamics should be targeted in future interventions to facilitate the effective spousal relationship and promote the wellbeing of carers.

## Methods

### Study design

This study used an interpretative phenomenological approach (IPA), following the guidelines of Smith and colleagues (2022). The primary aim of this study was to understand the complex, subjective experiences of family carers for people living with dementia, specifically focusing on the phenomena of interpersonal and intrapersonal dynamics. IPA allows to explore how individuals make sense of their lived experiences from their own perspectives. Thus, this analytical approach, which aligns with our aim, was used. Qualitative data were obtained through semi-structured interviews. This study was approved by the Faculty of Medicine and Health Sciences Research Ethics Subcommittee (FMH S-REC) of the University of East Anglia (ETH2122-0759). To ensure trustworthiness, this study applied the Lincoln et al. (1985) criteria, encompassing credibility, transferability, dependability, and confirmability. To determine dependability, the Standards for Reporting Qualitative Research (SRQR) were followed (O'Brien et al., 2014).

### Participant selection

Purposive sampling was used to recruit family carers who had the capacity to consent for themselves, were female, were an unpaid carer with a partner/spousal relationship, were caring for a community-dwelling person living with dementia, and were supporting activities of daily living of the care recipient on a day-to-day basis (i.e. more than 7 hours per week). Participants with an insufficient understanding of English to complete interviews were excluded. An invitation to the study was sent to potential participants who took part in other ethically approved dementia carer studies led by the authors and had consented to be further contacted about participation in other studies. Potential eligible participants were contacted by the researcher via telephone or email, depending on the carer’s preference, to check if they were happy to receive information about this study and to double check eligibility. Recruitment took place between March and July 2022. In total, nine family carers agreed to take part in the interview session.

### Data collection

After obtaining written informed consent, family carers were asked to complete a demographic questionnaire to characterise the sample. The interview session was conducted face-to-face in the participant’s own home or remotely via telephone or video call (Microsoft Teams). The semi-structured interviews lasted about 1 hour and were conducted by one researcher (EVH), a nurse with MSc in Human Sexuality Studies and experienced in qualitative research. The interview guide containing a list of questions and prompts was piloted with a family carer and revised until consensus between researchers was reached. The interview guide for the study is presented in online supplementary file 1. A state of not knowing and a blended approach were applied during the interview, which consists of passive interviewing (allowing the participant space and time to share their narrative) and more active approaches by using questions and prompts listed in the interview guide. After each interview, participants were offered debriefing sessions to ensure their wellbeing and provide an opportunity for reflection and discussion. Audiotaped interviews were transcript verbatim. The data collected were anonymised and checked for accuracy. Data collection continued until data saturation was attained, meaning that conducting additional interviews no longer provided new insights.

### Analysis

The questionnaire data was analysed descriptively to characterise the sample. Interview data were analysed following the IPA procedures, which involves several key steps. Specifically, IPA includes (1) familiarisation with the data through multiple readings of transcripts, (2) identification of meaning units reflecting how participants describe their experiences, (3) clustering of themes by grouping related meanings, and (4) development of comprehensive descriptions of the dynamics within and across individual experiences (Howitt & Cramer, 2011). To ensure analytical rigor, two researchers (EVH and MC) reviewed the transcripts independently. Both researchers had extensive involvement with the participants, stemming from prior experiences with the sample group. In addition, formulations of meanings and associated themes were generated by both coders. Any disagreement was settled through discussion between EVH and MC, with close supervision of NK. Analysis of all the individual transcripts was finalised before moving to the group-level analysis. A summary list of themes was compiled and related themes were clustered together at the group level. After creating a summary list, the themes were then coded onto all the transcripts using NVivo, providing the opportunity to check the accuracy of the themes. The retraction of those themes and corresponding quotes formed the basis for the account presented here.

## Results

Nine participants were interviewed between March and July 2022. Characteristics of participants are summarised in Table 1. The female carers had a mean age of 69.56 years (SD = 7.54) and cared for their male spouse or partner with a mean age of 75.44 (SD = 7.78). On average, participants had been providing care for 6.4 years (SD = 3.25). Findings from the data analysis are summarised by describing the impact of dementia caregiving on these four interpersonal and intrapersonal dynamics (i.e. relationship adjustment, emotional connection, communication engagement, sense of self) and how participants overcame difficulties associated with such changes.

**Table 1.** Demographic Characteristics.

	Frequency	Percentage %
Carer’s age		
60–69 years	5	55.56
70–79 years	3	33.33
80–89 years	1	11.11
Carer’s gender		
Female	9	100
Carer’s relationship to partner with dementia		
Spouses	9	100
Age of partner living with dementia		
60–69 years	2	22.22
70–79 years	4	44.44
80–89 years	3	33.33
Length of care		
0–4 years	3	33.33
5–9 years	5	55.56
10–14 years	1	11.11
Time since diagnosis (in years)		
0–4 years	1	11.11
5–9 years	7	77.78
10–14 years	1	11.11
Dementia diagnosis		
Alzheimer’s disease	3	33.33
Mixed dementia	3	33.33
Vascular dementia	1	11.11
Lewy body dementia	1	11.11
Early onset dementia	1	11.11

### Relationship adjustment

#### Impact of dementia on the roles within the relationship

Many participants expressed that they experienced gradual changes in the relationship between them and the person they were caring for. They particularly experienced those changes as losses, which became more evident when participants reflected on their own past relationship or when they compared themselves to other couples. All participants mentioned that their caring responsibilities were increasing while their husbands’ independence decreased. They described these increased responsibilities as “*being on call 24/7*” (Participant 9) and “*never having any downtime*” (Participant 10). The decreasing husband’s independence also involved a reduction in initiative and decision-making. Therefore, participants became the metaphorical driver of all decisions in their husbands’ lives.

“He is sort of more dependent on me, maybe, then otherwise. And he never… he does not really do anything, I guess, without me sort of organising it.” (Participant 1)

“I kind of took over and became the dominant partner. Well at the same time trying to keep his role as the man.” (Participant 10)

These changes in the husband’s independence due to the dementia symptoms also changed the roles within the relationship. Most participants expressed that they felt more like a carer rather than a companion towards their husband. To a great degree, participants were associating the caregiving skills they use with the parental skills of mothers when describing them. For example, participants were using metaphors to explain their husband’s behaviour referring to them as if they were raising a child during interviews.

“I talked to him like he is a child. I said, “why don’t you wear these and that would be really good for us” and then just put them on. He went, “oh, that’s great”. So now this is completely not romantic, it is fully carer.” (Participant 10)

“A bit like when you have children, they always want something when you're in the middle of relaxing. People always say I just sat down and the baby cried, or I just sat down and … it’s a similar thing, I think.” (Participant 8)

When asked about how this carer role was perceived within their relationship, two perceptions were mentioned. Some participants perceived their carer role as a marital duty, while others saw it as an act of love.

“This is about sort of ‘for better or worse’. It could have been the boot on the other foot. For some reason it is so. So I guess there is a kind of duty element here.” (Participant 5)

“When you have been with somebody most of their life, because I was young when I got married, that person is part of you in a way. […] They are literally the expression ‘your other half’. That is what he is. He is my other half” (Participant 4)

#### Adjusting to the changes in the relationship

The ability to adjust the relationship according to the “*good*’ and the “*bad*’ days (Participant 6) was highlighted as a critical element to overcome the relationship challenges. Participants described how certain methods worked one day, but on other days, those same methods would not work. This taught them to seize the good days whenever they occurred. In addition, participants highlighted the benefits of learning to step back when the behaviour of their husbands was challenging. They learnt that not all arguments and/or discussions with their husbands are worth pursuing, particularly when the behaviour displayed was repetitive.

“I guess if I think about it, I probably do mind but I think I just accepted it part of where we are really. Just sort of try and enjoy what we have, really.” (Participant 5)

“I knew [Husband] was, by lack of a better word, pushing my buttons this morning. So, I am not fighting you. So, I just literally turned my back on him and got on with something” (Participant 2)

### Emotional connection

#### Impact of dementia on emotional connection

Participants described how dementia led to changes in the emotional connection with their husband. They expressed how their husband changed their expression of love or compassion towards them. Some participants expressed how their husbands were more emotionally dependent on them than before. Other participants described how their husbands were less attentive towards them. Most participants reported that they had to grieve for the husband and the future they lost.

“I fell the other day because I was rushing around in the kitchen. I slipped and he did not realise that I had fallen and since then he has not asked me if I am okay or anything. He does not ask me if I am alright, whereas he would have done that before.” (Participant 6)

Some participants also described the ambivalent feeling of anticipatory grief (i.e. grieving the different losses associated with changes that occur before the death of a significant person) and happiness about still being able to have their husband with them.

“It is a double-edged sword, because I keep saying I am happy looking after [Husband], but I know he is dying. So I am not happy, I am probably in, I mean, permanent grief. All the time I am talking to you, I am nearly crying, because… But I am like that all the time, not just when I am talking to somebody. So, I am permanently in grief at the same time as I am really happy that I still have him with me.” (Participant 10)

#### Facilitating emotional connection in the relationship

Different strategies to facilitate the emotional connection were highlighted during interviews. Some participants mentioned that the emotional connection to their husband increased when there were moments of recognition. Meaning, the husband would recognise the carer or the memories they have created together. This connection through previously shared moments was generated by looking through pictures of their families or holidays and revisiting activities or places they have been in the past.

“I could see that he was not even sure who I was, and he had no idea who the grandchildren were… About half an hour into the drive home, suddenly he grabbed my arm, “you are [Carer’s name], you are my [Carer’s name]”, and there you go. That is the connection.” (Participant 10)

Participants also described how they became more observant of their husbands’ new ways of showing affection. This ranged from noticing their husbands physical touch (e.g. putting their hand on the carer’s back) to realising that their husbands are giving them space (e.g. going to a different room when carer is about to get angry). Compassion for their husbands’ struggles was also expressed by the participants. They were trying to become more empathetic and more sensitive towards their needs.

“I think sometimes he's aware of how I'm feeling because… sometimes he says “I think I'll just go in the next room now” because he can see I'm getting stressed out” (Participant 3)

“He is always sort of… very close and he always wants to hold my hand. We still sleep together, and we still share the same bed. He still cuddles up to me and that type of thing, you know. He wants to know that I am there.” (Participant 6)

### Communication engagement

#### Impact of dementia on the communication engagement

All participants expressed a loss of meaningful communication with their husband. Their conversations felt unproductive, and the content of the conversation was often not fully understood. This meant that participants were constantly feeling that their messages to their husbands did not fully get through, and that the comments made by their husbands were often hard to grasp for the participants, as they were not related to the context of the conversation. A few participants expressed how this led to them sharing less with their husband.

“He understands. But I do not tell him that much because it is not… well he does not really get everything. So, no, I do not really share everything with him like I used [to].” (Participant 6)

In addition, participants reported a loss of a two-way interaction when asked about their current communication engagement. Some participants described a rather silent life with a limited amount of small talk, while other participants described talking continuously without necessarily getting a response from their husband.

“There is barely no engagement from him. I am literally at no point even asking him a question. Let us say, “do you want a cup of tea?”. I just put the tea there and say, “There is the tea you wanted darling” and then he is so happy.” (Participant 10)

#### Dealing with the loss of communication engagement

Many participants expressed that knowing their husbands for a long time helped them to overcome the difficulties they were experiencing in terms of communication. Their understanding of their husbands helped the participants to deduce the content of the conversation even from a few words and recognise the complex feelings poorly expressed by their husbands.

“If you know the man you are married to, you do learn very quickly how to… when to stop or what to say and what matter you say it.” (Participant 9)

“I suppose in the previous three years, we'd already got to know each other so well, that I understood what he would want all the time..” (Participant 10)

Moreover, participants expressed that they watched their husband more closely and tried to learn their ways of communicating and read their signs. These strategies helped them to understand what their husband would need from them by reading signs unique to their husband, which are difficult to explain to others. Participants acknowledged that they became their husbands’ “*translator*” when speaking to others.

“You do learn things. For instance, I have learned to read the signs… particularly I can notice like if he wants to go to the toilet. I can notice that, and I have learned… I have learned to read that sign. So, you learn, and you cannot explain it to anyone else.” (Participant 2)

“If I am with him, they sort of look at me and say, “can you help me I don’t know what he’s doing or saying?”. And I think [Husband] relies on me to explain what his… he would feel very lost if he was trying to talk and I was not there.” (Participant 2)

### Sense of self

#### Impact of caregiving on the sense of self

When asked about how caregiving had affected their sense of self, participants expressed both positive and negative consequences. Some participants felt that caregiving made them more aware of other people’s circumstances, and consequently, allowed them to empathise more with others and have a better understanding of what they are going through. Other participants expressed that they had to become more tolerant and patient towards the repetition demonstrated by the person living with dementia and saw this as a positive change in the way they perceived themselves. Nevertheless, most participants mentioned feeling a loss of self and being “*trapped*” (Participant 5). They expressed a loss of activities and leisure time that defined themselves, due to an increase in responsibilities and caregiving duties, led to an increased sense of loss of self. There was limited room for their own lives, with participants describing a necessity to put their husbands’ needs before their own.

“It has taken away things from me. It has taken away some of my personality. It has taken away my thinking.” (Participant 9)

“Because I'm caring so much, my real self has been put on the shelf.” (Participant 10)

“Sometimes I feel like… You've probably seen those pictures of a person in prison with a chain and a big metal ball at the end of it.” (Participant 3)

Participants also voiced how the loneliness of caring made it harder for them. They felt misunderstood by people in their surroundings and healthcare professionals. In addition, participants expressed that their circle of friends and family decreased. This, in combination with limited free time, created a feeling of isolation and loss of self for the participants.

“I’m his sole care. So I can't say, “oh look, I've had enough. Could you take care of him today please?”. Because there isn't anyone else.” (Participant 3)

“I guess I feel I'm a bit… You are a bit on your own because I don't think people quite know what it's like.” (Participant 1)

#### Strategies to avoid loss of self

Some participants mentioned that the active search for support and understanding from others helped them to overcome the feeling of isolation and loss of self. Talking with other people facing similar situations and being able to “*unload*” (Participant 2) feelings was seen as beneficial for most participants. Some participants also mentioned that they found it particularly helpful to talk about something completely different. These participants felt relieved to not talk about their caregiving situation and the dementia progress.

“It is about being able to just unload and saying, “you know what, at the moment, I’m feeling really shit”. But that is a hard thing to say to people, unless they actually asked you.” (Participant 2)

“I value the relationship I have with the rest of my family, although they are not close enough to be able to help day to day. I think they understand the situation.” (Participant 8)

Furthermore, participants described techniques which helped them to be more kind to themselves. This was mainly achieved by focusing on the present task at hand (e.g. doing crossword) or by changing their mindset (e.g. not being too self-demanding or self-critical). Most participants also expressed the need for time for themselves. Participants mentioned that they were aware of non-negotiables (i.e. things and activities that are important to them) in their life that needed to be maintained, and that prioritising these, when possible, consequently allowed them to keep some of their own identity. However, half of those participants also mentioned feelings of guilt to leave their husband behind to prioritise their own needs.

“I do try to go out quite a lot. I hope I do. I am quite active because that keeps me going. But then I feel a bit guilty if I am leaving him.” (Participant 1)

“I try to do my normal things as well, to keep me balanced. Of course, I had to give up most of them. I still got a few, which I kind of called ‘my non-negotiables’.” (Participant 10)

“I still go and play badminton because I put my foot down on that. Because I can come home the same day and gosh I need some time for myself, don't I?” (Participant 3)

## Discussion

This study examined the subjective experiences of female spousal carers of people living with dementia, investigating both interpersonal (relationship adjustment, emotional connection, communication engagement) and intrapersonal (sense of self) dynamics, and carers’ strategies for adapting to the changes in such dynamics.

In the first theme ‘Relationship adjustment’, female spouses highlighted a critical change in their relationship, a decrease in initiative and decision-making demonstrated by their partner whereby carers became the metaphorical driver in their relationship. This is consistent with a systematic review highlighting the imbalance of the spousal relationship as dementia progresses (Pozzebon et al., 2016). Equity theory suggests that when the imbalance of the spousal relationship occurs in the context of non-dementia caregiving couples tend to strive to maintain a good balance of give and take in carer-partner relationships (Hatfield & Rapson, 2012; McPherson et al., 2011). However, strategies to maintain such balance was not evident in our sample potentially due to the severity of dementia. Instead, carers were acknowledging notable role changes, with the carer taking a more parental role although this caused a decrease in emotional connection. Qualitative study of Eskola and colleagues (2022) support this trend, as spousal carers described themselves as mothers. Carers were adjusting to these changes in the relationship by learning to step back when difficult moments arose, such as repetitive behaviour from their partner, and to be more present in the moment. This is consistent with systematic review on spousal dementia relationships highlighting ‘embracing a day-by-day approach’ as a key perspective for couples living with dementia (Stedje et al., 2023). During analysis, an overlap was identified between the theme of ‘relationship adjustment’ and other themes including emotional connection, communication engagement, and sense of self. This may suggest a potential interdependence, indicating that positive experiences within these themes may contribute to improved relationship adjustment among spousal carers. Future research could explore the potential overlapping impact of different aspects of the relationship on the wellbeing of carers. This exploration could utilise a methodology that allows to integrate both qualitative and quantitative approaches, such as Qualitative Comparative Analysis, to provide a comprehensive understanding of these dynamics.

Regarding the second theme ‘Emotional connection’, carers in this study expressed that they experienced significant losses in their relationships (i.e. anticipatory grief) and how their partner living with dementia changed their expression of love and compassion towards them affected emotional connection between them. This is consistent with a recent study examining the meaning of dementia for emotional connection (Eskola et al., 2022) and a systematic review on relationship changes in dementia (Conway et al., 2018), which highlighted how the lack of reciprocity of affection in couples weakened the physical and emotional intimacy. The findings of the current study demonstrated that carers were coping with such emotional challenges by reminiscing on their shared history. This reminiscing helped carers to feel connected with their partner and consequently maintaining a sense of shared identity as a couple.

The lack of reciprocity was also seen in the third theme ‘Communication engagement’. A loss of meaningful communication and loss of a two-way interaction was expressed by all carers in this study. This loss of two-way interaction and disturbed communication has been described in other literature as the greatest challenge in spousal dementia caregiving (Pozzebon et al., 2016). The findings of the current study demonstrated that carers were overcoming a lack of verbal communication engagement by watching their partner more closely and relying more on non-verbal ways of communicating, such as reading signs unique to their partner. Furthermore, carers appeared to recognize the necessity of embracing one-way communication.

In the last theme ‘Sense of self’, female spousal carers reported that they experienced a loss of self and isolation, stemming from a reduction in participating in activities and leisure time. This, combined with diminished external support, led to a feeling of loneliness. This phenomenon resonates with previous research, which highlights the tension resulting from greater partner dependency and diminishing carers’ inability to pursue their own activities, affecting carers’ sense of self (Tuomola et al., 2016). Similarly, Conway and colleagues (2018) highlighted in their systematic review how essential preserving their own identity is to carers. Interestingly, the carers in this study demonstrated a proactive stance in seeking support from various sources, including healthcare professionals, to overcome these challenges. This contradicts findings from prior research where family carers were often hesitant to ask for support due to fear of misunderstanding and sense of duty (Messina et al., 2022). In addition to seeking formal support, the findings of the current study demonstrated the importance of the carers’ ability to be self-compassionate towards oneself in maintaining their sense of self. This consists with a recent study which demonstrated that individuals who exhibit self-compassion while caring for people living with dementia are more inclined to exhibit improved psychological wellbeing (Lloyd et al., 2019).

### Clinical implications

There is an increasing interest in the significant effect dementia symptoms have on spousal relationships and the carers’ wellbeing (Hawken et al., 2018). However, to the best of our knowledge, this study is the first study to explore such impacts and highlight strategies that are potentially effective in overcoming relationship challenges using a qualitative approach. These findings revealed some effective strategies carers use to adjust to the changes in their relationship. These included acknowledging the role changes while stepping back when difficult moments arose, focusing on the present, reminiscing on their shared history, learning new ways of communicating (e.g. watching their partner more closely and relying more on non-verbal ways of communicating), and increasing self-compassionate attitudes towards themselves.

A recent scoping review of psychosocial interventions to enhance the relationship of couples living with dementia concluded that there was a limited body of evidence. This was partly due to the fact that studies included in the review did not provide a detailed account of the processes whereby the intervention was expected to benefit the relationship (Gilbert et al., 2023). Currently, single-component interventions such as cognitive behavioural therapy (CBT) are often used to improve psychological wellbeing, carer burden and relationship problems within family carers of people with dementia (Population Health et al., 2022). However, our findings suggested that improving the relationship between female spousal carers and their partner living with dementia may require targeted interventions addressing different factors.

There are some existing interventions that may be able to target factors identified in this study. One such an example is narrative therapy (i.e. couples life story approach or reminiscence therapy), which may be particularly beneficial for increasing emotional connection between the carer and their partner living with dementia (Gilbert et al., 2023). The ‘couple’s life story approach’ enables couples to reminisce about their shared experiences and shows to be an effective method for enhancing the quality of a couple’s relationship (Colloby et al., 2022). The process of reminiscing not only fosters the emergence of fresh insights concerning their partnership but also enhances comprehension of their caregiving responsibilities, even in situations where the dynamics of their relationship were imbalanced prior to the onset of dementia (Ryan et al., 2020). Nevertheless, while reminiscing can foster deeper emotional connection, it can also serve as a reminder for carers of the losses they have endured due to their partner’s dementia progression (Scherrer et al., 2014).

The use of interactive communication interventions with a focus on non-verbal ways of communicating may also be beneficial for improving communication engagement. These interventions are shown to have some preliminary effects on the communication and behavioural management skills of carers in their interactions with people living with dementia (Nguyen et al., 2019). A recent systematic review highlights the impact of communication partner training on facilitating relationships among family members of people living with dementia (Folder et al., 2023), with one feasibility study showing improvements in spousal relationship quality (Berwig et al., 2020).

Lastly, aspects of compassion-focused therapies, which promotes individuals to be compassionate toward themselves and other people (Gilbert, 2009), may be helpful to aid the relationship adjustment of carers and their partner with dementia. Compassion-focused therapy has shown to have positive effects on quality of life of couples living with dementia (Collins et al., 2018), and higher levels of self-compassion have been associated with more positive relationship behaviours as opposed to those with lower self-compassion (Neff & Beretvas, 2013). However, to date, no research has directly looked at the effectiveness of compassion-focused therapies on relationship related outcomes. Future studies are thus needed to develop these multifaceted interventions and evaluating their effectiveness on the quality of relationship of couples living with dementia.

### Limitations

While it may serve as a strength to focus on female carers, considering the predominance of female carers in the population, further studies should explore the impact of relationships on carer anxiety in male family carers to understand the role gender may play in long-term intimate relationship. This study solely focused on the experiences and perspectives of carers and the reciprocal nature of the relationship was not assessed. Future studies could employ a dyadic approach, accounting for the perspective of the person living with dementia. Furthermore, studies suggest ethnicity and culture may affect psychological wellbeing and their proactive stance in seeking support (Duran-Kiraç et al., 2022). The views of carers from minority ethnic groups on the relationship and the associated changes may thus be different. Participants in this study were recruited in the East of England where more than 90% of the population is White British, questions about ethnicity were not directly asked during the recruitment and interview process. Future studies should therefore aim to investigate a more diverse sample by including male spousal carers and spousal carers from minority ethnic groups.

## Conclusion

This study provided valuable insights to the growing body of knowledge on dementia caregiving relationships and demonstrated a deeper understanding of the meaning derived from interpersonal and intrapersonal dynamics and how carers adapt to changes in such dynamics. The findings highlighted the need for implementing diverse of interventions tailored for female spousal carers in maintaining and improving their relationship with their partner living with dementia. Such interventions could include a couple’s life story approach to enable couples to reminisce about their shared experiences, interactive communication training to enhance meaningful engagements, and a psychological approach such as compassion focused therapy to overcome emotional challenges and improve self-compassion. Future research should focus on developing and evaluating these interventions and explore whether different components can enhance the relationships of couples living with dementia, ultimately improve carers’ wellbeing.

## Supplemental Material

Supplemental Material - Understanding the impact of dementia on spousal relationships: A qualitative study with female spousal carers of people living with dementia Supplemental Material for Understanding the impact of dementia on spousal relationships: A qualitative study with female spousal carers of people living with dementia by Elien Van Hout, Milena Contreras, Eneida Mioshi, and Naoko Kishita in Dementia.
