# Supplementary material for: Understanding the impact of dementia on spousal relationships: A qualitative study with female spousal carers of people living with dementia
Source: Dementia (London). 2024 Sep 27;24(1):23–39. doi: 10.1177/14713012241286559 (PMC11667960; doi:10.1177/14713012241286559)
Supplement: Supplemental Material - Understanding the impact of dementia on spousal relationships: A qualitative study with female spousal carers of people living with dementia [file sj-pdf-1-dem-10.1177_14713012241286559.pdf]

Supplemental Material

Supplementary file 1: Interview guide

To start, could you briefly explain to me your own situation and that of your partners? I understand you are a carer for your partner, what does this entail for you?

Q1: Could you tell me what your role as a carer means within your relationship with your partner?

- *Possible prompt: Could you describe to me how this feels for you?*

Q2: To what extent do you feel your relationship has adjusted to the dementia diagnosis?

- *Possible prompts: How do you notice this? Could you give me some examples?; Could you describe to me how you can see your relationship has adjusted/changed? How important do you feel this relationship adjustment was for you in your relationship with your partner?*

Q3: How does the current caregiving situation affect your feelings towards the relationship?

- *Possible prompts: How do you currently view your relationship?*

Q4: Do you currently feel connected to your partner?

- *Possible prompts: How do you experience this connection to your partner?; Are there things that have complicated this connection throughout the disease process or the care for your partner? Could you explain to me the situations where this happened?; How important do you feel this connection is in your relationship with your partner with dementia?; Could you describe a situation where you felt connected with your partner? Could you describe the reason why you felt connected at that point?*

Q5: How do you feel the communication between you and your partner currently transpires?

- *Possible prompts: Can you describe to me how this makes you feel?; To what extent do you experience difficulties in communicating with your partner?; How do you overcome these challenges?*

Thank you for answering all these questions already. We’ve talked about how you experience the relationship to your partner; the last question is a bit more specific about yourself and your identity as a carer.

Q6: To what extent do you feel the dementia disease and the caregiving situation has an influence on who you are?

- *Possible prompts: How do you perceive yourself?; Do you feel you as a person have changed throughout this process? In which way (positive/negative)?; To what extent do you think this has an influence on how you currently feel?*

This is the end of the interview. Before I stop the recording…

Q7: Do you have any other thoughts on the topics we have discussed that you would like to share with me?

Q8: Do you have any questions you would like to ask me?

I would like to say thank you again for giving up your time to speak with me today. [DEBRIEF]
